# Mitochondrial Division Inhibitor 1 (mdivi-1) Protects Neurons against Excitotoxicity through the Modulation of Mitochondrial Function and Intracellular Ca^2+^ Signaling

**DOI:** 10.3389/fnmol.2018.00003

**Published:** 2018-01-17

**Authors:** Asier Ruiz, Elena Alberdi, Carlos Matute

**Affiliations:** ^1^Laboratorio de Neurobiología, Departamento de Neurociencias, Universidad del País Vasco (UPV/EHU), Bilbao, Spain; ^2^Laboratorio de Neurobiología, Centro Vasco Achucarro de Neurociencia, Zamudio, Spain; ^3^Laboratorio de Neurobiología, Centro de Investigación Biomédica en Red sobre Enfermedades Neurodegenerativas, Madrid, Spain

**Keywords:** mdivi-1, Drp1, calpain, calcium, mitochondria, NMDA, excitotoxicity

## Abstract

Excessive dynamin related protein 1 (Drp1)-triggered mitochondrial fission contributes to apoptosis under pathological conditions and therefore it has emerged as a promising therapeutic target. Mitochondrial division inhibitor 1 (mdivi-1) inhibits Drp1-dependent mitochondrial fission and is neuroprotective in several models of brain ischemia and neurodegeneration. However, mdivi-1 also modulates mitochondrial function and oxidative stress independently of Drp1, and consequently the mechanisms through which it protects against neuronal injury are more complex than previously foreseen. In this study, we have analyzed the effects of mdivi-1 on mitochondrial dynamics, Ca^2+^ signaling, mitochondrial bioenergetics and cell viability during neuronal excitotoxicity *in vitro*. Time-lapse fluorescence microscopy revealed that mdivi-1 blocked NMDA-induced mitochondrial fission but not that triggered by sustained AMPA receptor activation, showing that mdivi-1 inhibits excitotoxic mitochondrial fragmentation in a source specific manner. Similarly, mdivi-1 strongly reduced NMDA-triggered necrotic-like neuronal death and, to a lesser extent, AMPA-induced toxicity. Interestingly, neuroprotection provided by mdivi-1 against NMDA, but not AMPA, correlated with a reduction in cytosolic Ca^2+^ ([Ca^2+^]_cyt_) overload and calpain activation indicating additional cytoprotective mechanisms. Indeed, mdivi-1 depolarized mitochondrial membrane and depleted ER Ca^2+^ content, leading to attenuation of mitochondrial [Ca^2+^] increase and enhancement of the integrated stress response (ISR) during NMDA receptor activation. Finally, lentiviral knockdown of Drp1 did not rescue NMDA-induced mitochondrial fission and toxicity, indicating that neuroprotective activity of mdivi-1 is Drp1-independent. Together, these results suggest that mdivi-1 induces a Drp1-independent protective phenotype that prevents predominantly NMDA receptor-mediated excitotoxicity through the modulation of mitochondrial function and intracellular Ca^2+^ signaling.

## Introduction

Ca^2+^ signaling through NMDA and AMPA receptors is critically involved in synaptic activity and plasticity, as well as development of brain circuits and neuronal survival (Ewald and Cline, 2009). However, overactivation of these receptors induces intracellular Ca^2+^ overload that eventually leads to excitotoxic neuronal death (Choi, 1992), contributing to acute disorders of the central nervous system (CNS) including stroke and traumatic brain injury as well as neurodegenerative diseases (Lewerenz and Maher, 2015). In acute insults to the CNS, excitotoxicity is mainly mediated by NMDARs (Li and Wang, 2016) and depending on the intensity of the insult and mitochondrial function it causes either early necrosis or delayed apoptosis, through bioenergetic collapse, activation of calpains, oxidative stress and release of mitochondrial pro-apoptotic factors (Arundine and Tymianski, 2004).

Mitochondrial fission is necessary for the generation of new organelles as well as for mitochondrial quality control (Youle and van der Bliek, 2012). In mammals, it is triggered by dynamin-related protein 1 (Drp1), which forms ring-like structures around the constriction points of dividing mitochondria (Smirnova et al., 2001). However, in contrast to its role in cell survival, Drp1-induced mitochondrial fragmentation contributes to the release of pro-apoptotic factors during apoptosis (Frank et al., 2001). Indeed, excessive mitochondrial fission is involved in the pathogenesis of several neurodegenerative diseases (Reddy et al., 2011) and therefore pharmacological inhibition of Drp1 has become a promising neuroprotective strategy. Mitochondrial division inhibitor 1 (mdivi-1) is a quinazonilone derivative that was reported to inhibit Drp1-dependent mitochondrial fission and Bax/Bak-dependent cytochrome *c* release during apoptosis (Cassidy-Stone et al., 2008). Used as a Drp1-inhibitor, mdivi-1 attenuated neuronal apoptosis in animal models of brain ischemia (Zhang et al., 2013; Wang et al., 2014) and epilepsy (Qiu et al., 2013; Xie et al., 2016), both *in vivo* and *in vitro*, and reduced oxidative stress and synaptic depression in a model of Alzheimer’s disease (Baek et al., 2017). However, very recent data strongly suggest that mdivi-1 modulates mitochondrial bioenergetics and ROS production through a Drp1-independent mechanism that may provide cytoprotection (Bordt et al., 2017).

Activation of NMDARs induces mitochondrial fission in neurons (Rintoul et al., 2003) but whether it is triggered by Drp1 or contributes to excitotoxicity is still a matter of debate. Mdivi-1 protects neurons against kainic acid (Kim et al., 2016) and glutamate excitotoxicity (Grohm et al., 2012), whereas NMDA-induced delayed mitochondrial fission and apoptosis was related to a downregulation of mitochondrial fusion, rather than to a Drp1-mediated fragmentation (Martorell-Riera et al., 2014). To elucidate the mechanisms underlying the neuroprotective activity of mdivi-1 against excitotoxicity, we have studied its effects on mitochondrial fission, neuronal survival, intracellular Ca^2+^ dynamics and mitochondrial function during excitotoxicity *in vitro*. We found that mdivi-1 depolarizes mitochondria and modulates intracellular Ca^2+^ signaling, providing robust protection against NMDA-induced excitotoxicity through a Drp1-independent mechanism.

## Materials and Methods

### Animals

All experiments were conducted under the supervision and with the approval of the Animals Ethics and Welfare Committee of the University of the Basque Country (CEEA, Comité de Etica en Experimentación Animal). All experiments were conducted in accordance with the Directives of the European Union on animal ethics and welfare. All possible efforts were made to minimize animal suffering and the number of animals used.

### Reagents and Plasmids

Neurobasal^®^ medium, B-27 supplement, antibiotic-antimycotic, calcein acetoxymethyl ester (calcein-AM), JC-1 and rhodamine 123 were purchased from Invitrogen (Barcelona, Spain). N-Methyl-D-aspartic acid (NMDA), mdivi-1, HBSS, glycine, poly-L-ornithine, glutamine, thapsigargin, tunycamicin, EGTA and FCCP were obtained from Sigma (St. Louis, MO, USA). α-amino-3-hydroxy-5-methyl-4-isoxazolepropionic acid (AMPA), kainate and cyclothiazide (CTZ) were obtained from Tocris Biosciences (Minneapolis, MN, USA). Cytotox 96^®^ for LDH release quantification was purchased from Promega (Madison, WI, USA). The plasmid expressing mitochondria-targeted Ca^2+^ indicator (2mtD4cpv) was kindly provided by Roger Tsien (University of California, San Diego, CA, USA). Lentiviral particles carrying a Drp1-shRNA vector were obtained from Santa Cruz Biotechnology (Dallas, TX, USA).

### Neuronal Primary Culture, Transfection and Lentiviral Knockdown

Cortical neurons were obtained from the cortical lobes of E18 Sprague-Dawley rat embryos according to previously described procedures (Larm et al., 1996; Cheung et al., 1998). Neurons were resuspended in 10% FBS-containing Neurobasal^®^ medium supplemented with B27, glutamine (2 mM) and antibiotic-antimycotic mixture, and seeded onto poly-L-ornithine-coated 48 well plates or glass coverslips (7 mm in diameter) at 1.5 × 10^5^ cells per well. For confocal single cell imaging experiments, cells were plated onto glass-bottom μ-dishes (Ibidi GmbH, Germany). The medium was replaced by serum-free, supplemented Neurobasal^®^ medium 24 h later. The cultures were essentially free of astrocytes and microglia and were maintained at 37°C and 5% CO_2_. Cultures were used at 8–10 days *in vitro* (DIV).

For transfection of cells, 4 × 10^6^ rat neurons were transfected in suspension with 3 μg of cDNA using Rat Neuron Nucleofector^®^ Kit (Lonza, Switzerland) according to the manufacturer’s instructions and plated and maintained as described above.

Drp1 knockdown was carried out by lentiviral delivery of expression constructs encoding target-specific shRNA (Santa Cruz Biotechnology). Neurons were infected at 2 DIV following standard procedures and treated with puromycin (1 μg/ml) from 4 DIV to 7 DIV for selection of cells expressing shRNA. For imaging experiments infected neurons were plated onto 7 mm glass coverslips in 48-well plates. Cultures were used at 9 DIV. All the procedures with lentiviral particles were performed in a biosafety level 2 (BSL-2) laboratory.

### Mitochondrial Fragmentation Analysis

Neurons expressing mitochondria-targeted 2mtD4cpv were exposed to agonists in Ca^2+^ and Mg^2+^-free HBSS containing 20 mM HEPES, 10 mM glucose, 10 μM glycine and 2.6 mM CaCl_2_ (incubation buffer) and z-stacks of the yellow fluorescent protein (YFP) were acquired through a 63× objective by inverted LCS SP2 or TCS SP8X confocal microscopes (Leica, Germany) at an acquisition rate of 1 stack/5 min during the indicated time period. To evaluate mitochondrial fission in neurons expressing 2mtD4cpv and lentiviral shRNA, neurons were fixed after treatment and YFP fluorescence was acquired through a Plan-Apochromat 20X/0.8 NA objective in an inverted widefield Zeiss Axio Observer microscope (Zeiss, Germany), equipped with an AxioCam MRm camera. After the time-lapse or cell fixation, number of cells with tubular and fragmented mitochondrial network was counted for data analysis.

### Cytosolic Ca^2+^ Imaging

Measurements of [Ca^2+^]_cyt_ were carried out as previously described (Ruiz et al., 2014). Neurons were loaded with Fluo-4 AM (1 μM; Molecular Probes, Invitrogen, Barcelona, Spain) in incubation buffer for 30 min at 37°C followed by 20 min wash to allow de-esterification. Images were acquired through a 63X objective by inverted LCS SP2 confocal microscope (Leica, Germany) at an acquisition rate of 1 frame/15 s during 5 min. For data analysis, a homogeneous population of 15–25 cells was selected in the field of view and neuronal somata selected as ROIs. Background values were always subtracted and data are expressed as *F*/*F*_0_ ± SEM (%) in which *F* represents the fluorescence value for a given time point and *F*_0_ represents the mean of the resting fluorescence level.

### Mitochondrial Ca^2+^ Imaging

Neurons transfected with mitochondria-targeted 2mtD4cpv Ca^2+^ indicator (Palmer et al., 2006) were transferred to incubation buffer (see above) and imaged by a TCS SP8X confocal microscope (Leica, Germany) as described before (Hill et al., 2014). Cells were excited at 458 nm and cfp and yfp emission aquired for FRET ratio quantification at an acquisition rate of 1 frame/15 s during 5 min. For data analysis, a homogeneous population of 5–12 cells was selected in the field of view and neuronal somata selected as ROIs. Background values were always subtracted and data are expressed as *R/R_0_* ± SEM (%) in which *R* represents the YFP/CFP fluorescence ratio for a given time point and *R*_0_ represents the mean of the resting FRET ratio.

### Toxicity Assays

In NMDA-mediated toxicity assays, neurons were exposed to NMDA in HBSS (free of Ca^2+^ and Mg^2+^) containing 2.6 mM CaCl_2_, 10 mM glucose and 10 μM glycine for 30 min at 37°C and washed with supplemented Neurobasal^®^. In AMPA-mediated toxicity assays, cells were stimulated with 25 μM of AMPA plus 100 μM cyclothiazide in supplemented Neurobasal^®^ for 30 min at 37°C and washed. Mdivi-1 was present 1 h before and during the excitotoxic insults and cell viability was assessed 1 h later by Citotox 96^®^ colorimetric assay (Promega, Madison, WI, USA) or 24 h later by fluorescent vital dye calcein-AM by in a Synergy™ H4 Hybrid microplate reader (BioTek, Winooski, VT, USA). All experiments were performed in quadruplicate and the values provided are the normalized mean ± SEM of at least three independent cultures.

### Western Blotting

Triplicates of 1.5 × 10^5^ cells were washed with PBS and harvested in 50 μl of ice-cold electrophoresis sample buffer. Lysates were boiled for 10 min, separated by electrophoresis using Criterion™ TGX™ Precast 12% gels and transferred to Trans-Blot^®^ TurboTM Midi Nitrocellulose or PVDF Transfer Packs (Bio Rad, Hercules, CA, USA). For immunoblotting, membranes were blocked in 5% skimmed milk, 5% serum in TBST and proteins detected by specific primary antibodies diluted in TBST containing 5% BSA overnight at 4°C: anti-αII Spectrin (1:1000; Santa Cruz Biotechnology); anti-PARP (1:1000, Cell Signaling, Danvers, MA, USA); anti-caspase-3 (Santa Cruz Biotechnology); anti-peIF2 and anti-eIF2 (1:1000; Cell Signaling); anti-KDEL (1:1000; Stressgen Bioreagents); anti-CHOP (1:250; Santa Cruz Biotechnology). After washing, membranes were incubated with horseradish peroxidase-conjugated secondary antibodies (1:2000, Sigma) in 5% skimmed milk, 1% normal serum in TTBS for 2 h RT and developed using enhanced chemiluminiscence according to the manufacturer’s instructions (Super Signal West Dura, Pierce, Rockford, IL, USA) in a C-Digit^®^ Blot Scanner (Li-Cor, Lincoln, NE, USA). Signals were quantified using Image Studio™ software (Li-Cor) and values were normalized to β-actin signal and provided as the mean ± SEM of at least three independent experiments.

### Mitochondrial Membrane Potential (∆Ψ_m_) Measurements

For quantification of mitochondrial membrane potential, neurons were loaded with quenching concentrations of rhodamine 123 (Rh 123, 5 μM) for 10 min followed by 20 min wash. Images were acquired through a 63× objective by inverted LCS SP2 confocal microscope (Leica, Germany) at an acquisition rate of 1 frame/15 s for 5 min. FCCP was added to depolarize the mitochondrial membrane and the increase in Rh 123 fluorescence was measured to estimate the ∆Ψ_m_. Data analysis was performed as described above (see “Cytosolic Ca^2+^ Imaging” section). Alternatively, cells were loaded with JC-1 dye for 15 min after the addition of mdivi-1 or FCCP respectively and red/green fluorescence ratio was measured by a Synergy™ H4 Hybrid microplate reader (BioTek, Winooski, VT, USA). All experiments were performed in quadruplicate and the values provided are the normalized mean ± SEM of at least three independent experiments.

### Measurement of Oxygen Consumption Rate

Oxygen consumption rate (OCR) was analyzed by a Seahorse XF96 Extracellular Flux Analyzer and XF Cell Mito Stress Test Kit (Agilent Technologies, Santa Clara, CA, USA) following manufacturer’s instructions. Neurons (3 × 10^4^ per well) were seeded on a poly-L-ornithine-coated XF96 plate and incubated in a modified ACSF containing (in mM) 126 NaCl, 3.0 KCl, 1.25 NaH_2_PO_4_, 2.0 CaCl, 10 glucose, 1.0 pyruvate, 2.0 glutamine and 0.01 glycine 1 h before the experiment. For the determination of basal, ATP-linked and maximal OCR during excitotoxicity three baseline recordings were made, followed by the sequential addition of NMDA or vehicle, oligomycin (2 μM), FCCP (1 μM) and rotenone/antimycin A (500 nM). To normalize OCR for cell viability changes during the experiment, LDH release was quantified at FCCP addition time point in parallel 48-well standard plates.

### Data Analysis

All data are expressed as mean ± SEM (*n*), where *n* refers to the number of cultures assayed, each obtained from a different group of animals. In single live cell imaging experiments, *n* refers to number of cells recorded from at least three independent cultures obtained from different groups of animals. For statistical analysis of the [Ca^2+^]_cyt_, [Ca^2+^]_mit_ and ∆Ψ_m_, basal line-extracted area under curve was calculated from single cell imaging time-lapse curves. Normality tests were carried out using GraphPad Prism software, and Student’s *t*-test or Mann-Whitney’s *U* test were applied for parametric and nonparametric tests, respectively. Statistical significance was determined at *p* < 0.05.

## Results

### NMDA-Induced Mitochondrial Fission Is Attenuated by Mdivi-1

**Figure 1:**
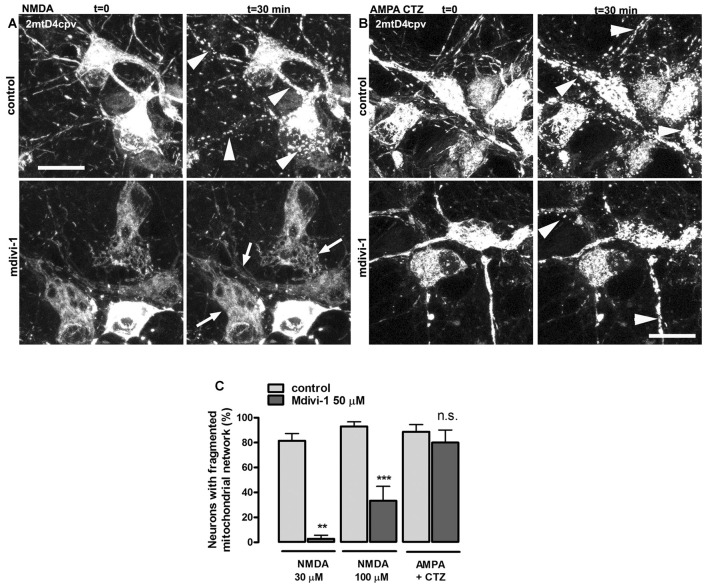
Mitochondrial division inhibitor 1 (mdivi-1) blocks NMDA but not AMPA/cyclothiazide (CTZ)-induced mitochondrial fission. **(A,B)** Representative time-lapse images of NMDA- and AMPA/CTZ induced mitochondrial fragmentation. Neurons transfected with mitochondria-targeted 2mtD4cpv were exposed to 30 μM or 100 μM of NMDA or 25 μM of AMPA plus CTZ (100 μM) for 30 min in the presence or absence of preincubated (1 h) mdivi-1 (50 μM). Arrows and arrowheads indicate representative neurons with non-fragmented and fragmented mitochondrial network respectively. Scale bar: 20 μm. **(C)** Mitochondrial morphology analysis from images acquired as described in **(A,B)**. Neurons were treated with NMDA at 30 μM or 100 μM in control conditions (*n* = 83 and 81, respectively) or in the presence of mdivi-1 (50 μM; *n* = 37 and 48, respectively). Mitochondrial network morphology was analyzed as well in neurons exposed to AMPA/CTZ in the absence or presence of mdivi-1 (*n* = 88 and 74, respectively). **p < 0.01, ***p < 0.001 compared to NMDA or AMPA/CTZ alone, Mann-Whitney *U* test.

NMDA receptor activation induces early and transient mitochondrial fission in neurons (Martorell-Riera et al., 2014). To analyze the effects of mdivi-1 on NMDA-induced mitochondrial fission, we exposed primary cortical neurons to increasing concentrations of NMDA in the presence or absence of pre-incubated mdivi-1 (50 μM, 1 h) and assessed mitochondrial network morphology of individual neurons by time-lapse microscopy. After 30 min exposure, 30 μM and 100 μM of NMDA triggered a dose-dependent mitochondrial fission in most of the neurons assayed (81.5 ± 5.7% and 93 ± 3.7%, respectively). In the presence of mdivi-1 the number of cells with fragmented mitochondrial network was strongly reduced to 2.8 ± 2.8% and to 33.4 ± 11.5% after incubation with NMDA at 30 μM and 100 μM, respectively (Figures 1A,C).

Next, we studied the effects of mdivi-1 on mitochondrial morphology after activation of non-NMDA glutamate ionotropic receptors such as AMPARs, since it was shown that KARs activation does not induce mitochondrial fission (Rintoul et al., 2003). Stimulation of neurons with 25 μM of AMPA in the presence of CTZ, to inhibit desensitization, markedly fragmented mitochondrial network in 88.7 ± 5.7% of treated cells. Strikingly, mdivi-1 did not inhibit AMPA/CTZ-induced mitochondrial fission (Figures 1B,C), indicating that it prevents selectively NMDA-induced early mitochondrial fragmentation.

### Mdivi-1 Protects Neurons against Excitotoxicity

**Figure 2:**
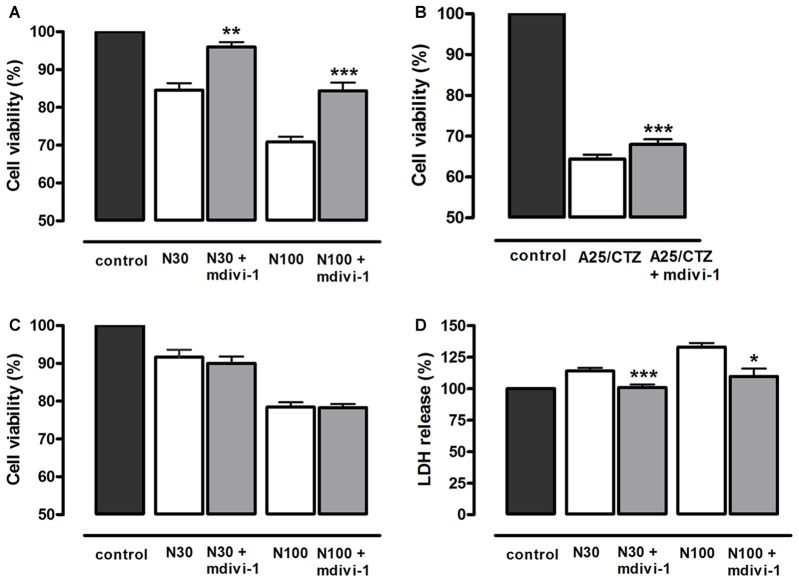
Mdivi-1 protects neurons from excitotoxicity. **(A,B)** Neurons were stimulated with **(A)** NMDA (30 μM and 100 μM, 30 min) or **(B)** AMPA (25 μM, 30 min) and (CTZ, 100 μM) in the presence or absence of mdivi-1 (50 μM, 1 h) and 24 h later cell viability was assessed by the quantification of vital dye calcein-AM fluorescence (*n* = 6 and *n* = 8, respectively). **(C)** Neurons were exposed to mdivi-1 (50 μM) after being stimulated with 30 and 100 μM of NMDA for 30 min, and 24 h later cell viability was assessed by the analysis of calcein-acetoxymethyl (AM) fluorescence (*n* = 3). **(D)** Neurons were stimulated with 30 μM and 100 μM of NMDA for 30 min in the presence or absence of mdivi-1 (50 μM, 1 h) and 1 h later LDH release to the extracellular medium was quantified (*n* = 4). Data represent means ± SEM of normalized calcein fluorescence values. *p < 0.05, **p < 0.01, ***p < 0.001 compared with control (NMDA or AMPA/CTZ alone), paired Student’s *t*-test.

In order to investigate the mechanisms involved in the neuroprotective action of mdivi-1, we first analyzed whether this inhibitor protected against toxic activation of NMDA and AMPA receptors. To that aim, cultured neurons were incubated with mdivi-1 (50 μM) before and during application of NMDA or AMPA/CTZ and cell viability was assessed 24 h later by calcein fluorescence analysis. NMDA at 30 μM and 100 μM induced a reduction in neuronal viability of 15.4 ± 1.82% and 29.1 ± 1.36% compared to control (untreated cells, 100%), that was attenuated by mdivi-1 to 3.9 ± 1.26% and 15.6 ± 2.1%, respectively (Figure 2A). On the other hand, AMPA (25 μM) in the presence of CTZ (100 μM) induced a decrease in neuronal viability of 35.5 ± 1% compared to control (untreated cells, 100%), and was moderately but significantly reduced by mdivi-1 to 31.9 ± 1% (Figure 2B). Next, we analyzed whether mdivi-1 protected neurons from excitotoxicity through the inhibition of either a delayed apoptotic event or an early necrotic cell death. Incubation of neurons with mdivi-1 after NMDA washing was ineffective against excitotoxicity (Figure 2C), whereas mdivi-1 preincubation attenuated LDH release as early as 1 h after NMDA (Figure 2D).

**Figure 3:**
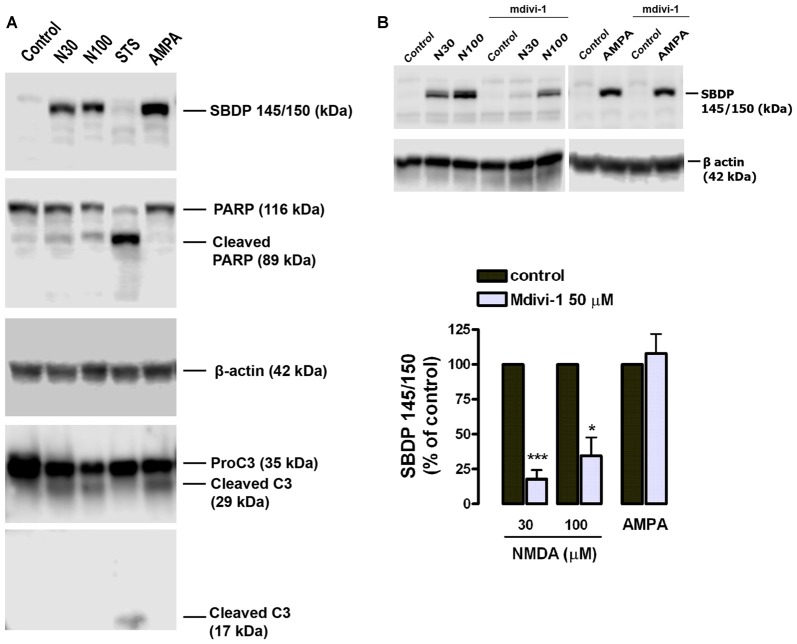
Mdivi-1 reduces NMDA-induced calpain activation. **(A)** Neurons were exposed to NMDA (30 μM and 100 μM, 30 min), staurosporine (STS, 1 μM) or AMPA (25 μM, 30 min) plus CTZ (100 μM) and harvested 24 h later for the detection of αII-spectrin breakdown products (SBDP), cleaved PARP, cleaved caspase-3 and β-actin by western blot. **(B)** Cells were stimulated with NMDA (30 and 100 μM, 30 min) and AMPA (25 μM, 30 min) plus CTZ (100 μM) with or without mdivi-1 (50 μM) and harvested 4 h later for the detection of 145 and 150 kDa SBDP. For the quantification of calpain activation, SBDP150/145 signal was measured and normalized to β-actin values. *p < 0.05, ***p < 0.01 compared with NMDA 30 μM (*n* = 5) and NMDA 100 μM (*n* = 4) alone or AMPA/CTZ (*n* = 3) alone, paired Student’s *t*-test.

Since calpains are major mediators of excitotoxic necrosis (Wang, 2000) we next examined whether mdivi-1 prevented their activation in this excitotoxicity paradigm. First, we confirmed that both NMDA and AMPA/CTZ led to activation of calpains rather than caspase-3 in cultured cortical neurons, as previously described (Ruiz et al., 2014). Excitotoxic insults generated αII-spectrin 145/150 kDa breakdown product (SBDP145/150), which is indicative of calpain activity (Moore et al., 2002), concomitantly with the cleavage of pro-caspase-3 into a 29 kDa fragment, also specific of calpain activation (Lankiewicz et al., 2000; Blomgren et al., 2001). In contrast, the classical apoptosis inducer staurosporine (STS) robustly induced caspase-3 break down into a 17 kDa fragment along with downstream cleavage of PARP into a 89 kDa product (Figure 3A). Next, neurons were exposed to NMDA in the absence or presence of mdivi-1 to analyze the levels of SBDP145/150. We found that 30 μM and 100 μM of NMDA induced a dose-dependent SBDP145/150 production that was drastically reduced by mdivi-1 to 17.6 ± 6.6% and 34.5 ± 15.3% of control (NMDA alone, 100%), respectively (Figure 3B). In contrast, mdivi-1 failed to attenuate AMPA/CTZ-induced αII spectrin cleavage as SBDP145/150 level were not altered (107 ± 14.2% of control using AMPA plus CTZ alone as 100%; Figure 3B). These results suggest that mdivi-1 protects against excitotoxicity predominantly by reducing NMDA-induced calpain activation and necrosis.

### Mdivi-1 Modulates Intracellular Ca^2+^ Homeostasis during Excitotoxicity

**Figure 4:**
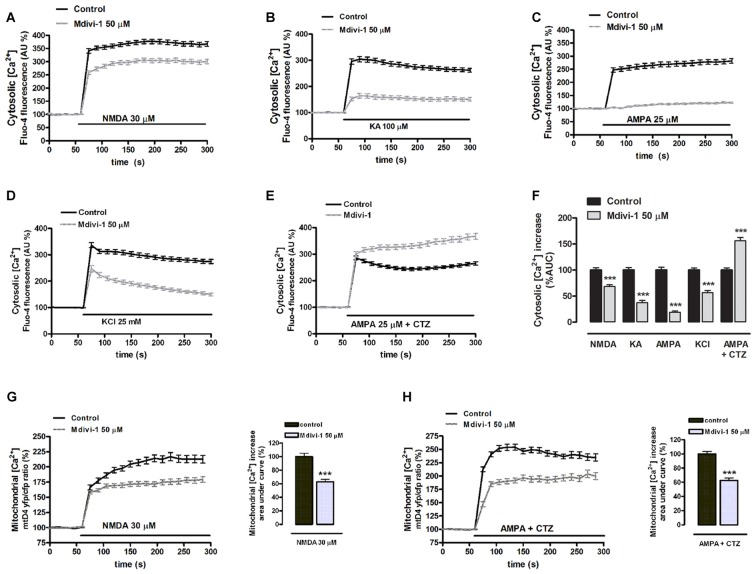
Mdivi-1 regulates cytosolic and mitochondrial Ca^2+^ overload during excitotoxicity. **(A–F)** Fluo-4-loaded neurons were exposed to NMDA (30 μM), kainate (KA, 100 μM), AMPA (25 μM), high [K^+^] and AMPA/CTZ as indicated in the absence or presence of mdivi-1 (50 μM, 1 h) and cytosolic Ca^2+^ load quantified. Traces represent normalized means ± SEM of more than 50 cells from at least three independent cultures/experiments. For statistical analysis normalized average ± SEM of the area under the curve was calculated. ***p < 0.001 compared to control cells (NMDA, KA, AMPA, 25 mM K^+^ or AMPA/CTZ alone); Mann-Whitney U test. **(G,H)** NMDA and AMPA/CTZ were added as indicated and mitochondrial Ca^2+^ levels monitored in the absence (*n* = 78 and *n* = 50 cells) or presence (*n* = 97 and *n* = 37 cells) of mdivi-1 (50 μM, 1 h). Traces represent the time course of normalized yfp/cfp average ratios ± SEM of cells from at least three independent culture/experiments. For statistical analysis, normalized average ± SEM of the area under the curve was calculated. ***p < 0.001 compared to NMDA alone (Mann-Whitney U test), or to AMPA/CTZ alone (Student’s *t*-test).

Because calpains are Ca^2+^-activated proteases and mitochondrial dynamics influence Ca^2+^ signaling (Szabadkai et al., 2006), we tested the hypothesis that mdivi-1 modulates intracellular Ca^2+^ fluxes during excitotoxicity. Neurons were exposed to agonists in order to separately activate different ionotropic glutamate receptors in the absence or presence of mdivi-1 and cytosolic Ca^2+^ levels ([Ca^2+^]_cyt_) were assessed by time-lapse fluorescence microscopy. Application of NMDA (30 μM), KA (100 μM) and AMPA (25 μM) induced a fast [Ca^2+^]_cyt_ increase with a peak amplitude of 367.7 ± 8.4%, 304.2 ± 10.2% and 282.1 ± 9% compared with resting levels (100%) that was reduced by 50 μM mdivi-1 to 306.0 ± 8.2%, 164.5 ± 9.3% and 123 ± 3%, respectively (Figures 4A–C,F). Together with glutamate ionotropic receptors, voltage gated Ca^2+^ channels (VGCCs) contribute to excitotoxicity (Prehn et al., 1995) and thus we tested whether mdivi-1 modified depolarization-induced [Ca^2+^]_cyt_ increase in neurons. Application of high [KCl] (25 mM, 5 min) induced a fast [Ca^2+^]_cyt_ peak of 336.5 ± 9.7% over basal levels (100%) that was attenuated to 247.3 ± 12.2% by 50 μM of mdivi-1 (Figures 4D,F). In contrast, in neurons treated with desensitizing AMPA (25 μM AMPA plus 100 μM CTZ) [Ca^2+^]_cyt_ increased to 286.4 ± 6.9% of resting levels and was further enhanced to 367.5 ± 11% in the presence of mdivi-1 (Figures 4E,F). We next analyzed whether the reduction of NMDA-induced [Ca^2+^]_cyt_ transients by mdivi-1 was a consequence of an enhanced mitochondrial Ca^2+^ uptake, since NMDAR shows a privileged access to mitochondria (Peng and Greenamyre, 1998). Neurons were transfected with a genetically encoded Ca^2+^ indicator (2mtD4cpv), exposed to NMDA or AMPA/CTZ and mitochondrial Ca^2+^ levels ([Ca^2+^]_mit_) assessed by time-lapse confocal microscopy. NMDA (30 μM, 5 min) induced a [Ca^2+^]_mit_ peak increase of 216.9 ± 6.7% compared to resting levels (100%) that was attenuated to 179.4 ± 4.7% by 50 μM of mdivi-1 (Figure 4G), suggesting that reduced cytosolic Ca^2+^ load in the presence of the Drp1 inhibitor is not due to an increased mitochondrial buffering. In AMPA/CTZ-stimulated neurons mdivi-1 reduced as well [Ca^2+^]_mit_ increased from 254.2 ± 5.6% to 196.5 ± 5.3% (Figure 4H).

**Figure 5:**
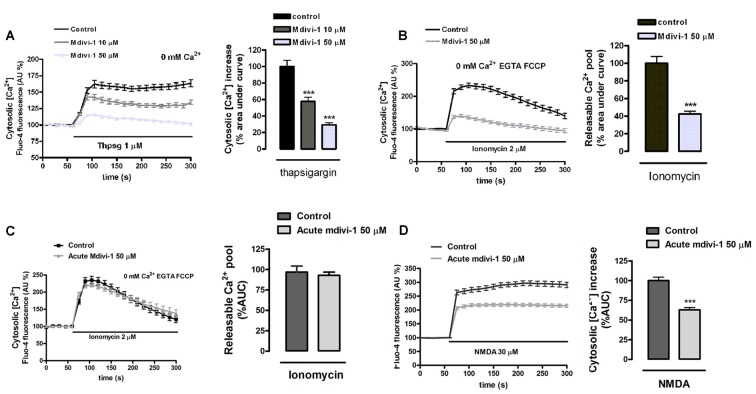
Preincubation of neurons with mdivi-1 depletes ER Ca^2+^ store. **(A,B)** Neurons were incubated with Fluo-4 in a Ca^2+^-free medium and exposed to **(A)** thapsigargin and **(B)** ionomycin (2 μM) plus FCCP (2 μM) in the presence or absence of mdivi-1 (50 μM). Resulting cytosolic Ca^2+^ increase was measured to determine ER Ca^2+^ content. **(C)** Ionomycin (2 μM) plus FCCP (2 μM) was added in the presence (*n* = 60 cells) or absence (*n* = 59 cells) of acute mdivi-1 (50 μM, 5 min). Resulting cytosolic Ca^2+^ increase was measured to determine ER Ca^2+^ content. **(D)** Fluo-4-loaded neurons were exposed to NMDA (30 μM) as indicated in the absence (*n* = 83) or presence (*n* = 90) of mdivi-1 (50 μM, 5 min) and cytosolic Ca^2+^ load quantified. Traces represent normalized means ± SEM of cells from at least three independent cultures/experiments. ***p < 0.001 compared to control cells (thapsigargin, ionomycin or NMDA alone), Mann-Whitney U test.

On the other hand, we previously reported that endoplasmic reticulum Ca^2+^ store contributes to [Ca^2+^]_cyt_ overload during excitotoxicity (Ruiz et al., 2009). To determine whether mdivi-1 regulated endoplasmic reticulum Ca^2+^ levels ([Ca^2+^]_ER_) we compared thapsigargin-induced [Ca^2+^]_cyt_ increase in control and mdivi-1-treated neurons. We observed that inhibition of SERCA pumps triggered a rise in [Ca^2+^]_cyt_ of 162.2 ± 5.8% of resting levels (100%) that was diminished by 10 μM and 50 μM of mdivi-1 to 142.4 ± 4.2% and 115.7 ± 1.6%, respectively (Figure 5A). Alternatively, we estimated [Ca^2+^]_ER_ measuring ionomycin-induced cytosolic Ca^2+^ signals in the absence of extracellular Ca^2+^ and after mitochondrial Ca^2+^ depletion by FCCP (Logan et al., 2014). We found that 50 μM of mdivi-1 strongly reduced ionomycin-releasable Ca^2+^ pool to 42.6% compared to control cells (100%; Figure 5B). However, acute addition of mdivi-1 (5 min incubation) did not affect ionomycin-releasable Ca^2+^ pool (Figure 5C) but reduced NMDA-induced [Ca^2+^]_cyt_ increased similarly to 1 h mdivi-1 exposure (Figure 5D), showing that the negative effects of the inhibitor on Ca^2+^ signals are independent of a Ca^2+^ induced Ca^2+^ release (CICR) from the ER. In summary, these results indicate that mdivi-1 regulates the [Ca^2+^]_cyt_ rise after activation of ionotropic glutamate receptors and VGCCs while reducing mitochondrial Ca^2+^ overload and basal ER Ca^2+^ content.

### Mdivi-1 Enhances NMDA-Activated Integrated Stress Response

**Figure 6:**
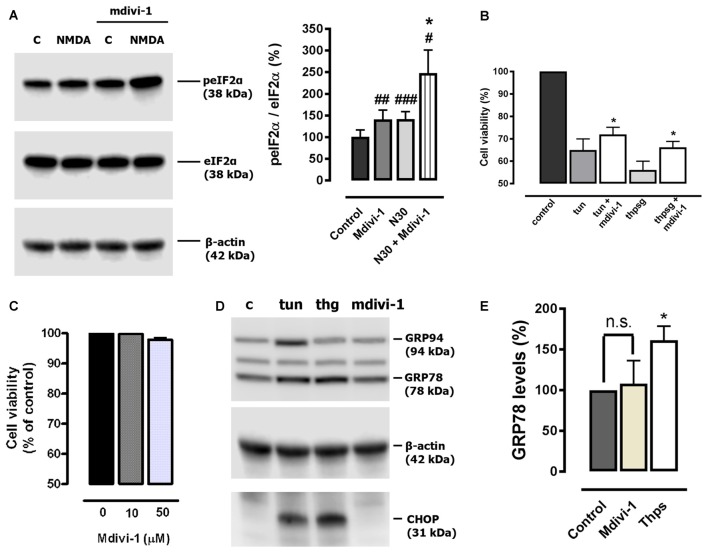
Mdivi-1 enhances the integrated stress response (ISR). **(A)** Neurons were stimulated with NMDA (30 μM, 30 min) with or without mdivi-1 (50 μM) and harvested. For the quantification of eukaryotic initiation factor 2 alpha (eIF2α) phosphorylation, peIF2α signal was measured and normalized to total eIF2α values (*n* = 6). ^#^*p* < 0.05, ^##^*p* < 0.01,^###^*p* < 0.001 compared with untreated cells; *p < 0.05, compared with NMDA-treated cells, paired student’s *t*-test. **(B)** Neurons were stimulated with 1 μM of tunicamycin (tun) or thapsigargin (thpsg) in the presence or absence of pre-incubated mdivi-1 (50 μM, 1 h) and cell viability was assessed 48 h later by calcein-AM fluorescence (*n* = 4). Data represent normalized means ± SEM. *p < 0.05 compared with control cells (tunicamycin or thapsigargin alone), paired Student’s *t*-test. **(C)** Neurons were incubated with mdivi-1 (10 μM and 50 μM) for 24 h and cell viability was determined by calcein-AM fluorescence (*n* = 3). Data represent normalized means ± SEM. Paired Student’s *t*-test. **(D)** Cells were exposed to 1 μM of tunicamycin (tun), thapsigargin (thg) or mdivi-1 (50 μM) and harvested 24 h later for the detection of GRP94, GRP78, CHOP and β-actin by western blot. **(E)** For quantification cells were treated with mdivi-1 (50 μM) or thapsigargin, and harvested 24 h later for the detection of GRP78 and CHOP (*n* = 3). Data represent normalized means ± SEM. *p < 0.05 compared with untreated control cells, Paired Student’s *t*-test.

Disruption of intracellular Ca^2+^ homeostasis leads to the induction of the UPR (Krebs et al., 2015) and previous reports have shown that both events can take place during excitotoxic conditions (Sokka et al., 2007; Ruiz et al., 2009). Since mdivi-1 regulated ER Ca^2+^ homeostasis, we explored a possible effect of the inhibitor on the phosphorylation of the eukaryotic initiation factor 2 alpha (eIF2α), a fundamental component of the unfolded protein response (UPR) and the core of the ISR (Donnelly et al., 2013). Neurons were treated with 30 μM of NMDA in the presence or absence of pre-incubated mdivi-1 (50 μM, 1 h) and phosphorylation of eIF2α was analyzed by immunoblotting. NMDA triggered a peIF2α increase of 143.2 ± 9.9% of control (untreated cells, 100%) that was enhanced to 246.7 ± 34.1% in the presence of mdivi-1 (Figure 6A). Interestingly, mdivi-1 alone significantly increased as well the phosphorylation of eIF2α to 150.3 ± 16.4% of control.

Cytoprotection against ER stress is a previously described effect of pharmacological ISR potentiation (Tsaytler et al., 2011). Thus, to determine the relevance of the enhancement of the ISR provided by midivi-1 we tested its efficacy against classical ER stressors. We found that mdivi-1 reduced toxicity in neurons treated with tunycamicin and thapsigargin from 35 ± 5% to 28 ± 3.2% and from 43.8 ± 3.9% to 33.8 ± 2.6%, respectively (Figure 6B). Since mdivi-1 depleted ER Ca^2+^ and enhanced eIF2α phosphorylation, we reasoned that it could induce itself ER stress and the UPR in neurons. However, mdivi-1 alone did not induce neuronal death (Figure 6C) or upregulate either GRP78 nor CHOP expression, hallmarks of the UPR and ER stress-induced apoptosis respectively (Paschen and Mengesdorf, 2005). As experimental control, addition of tunycamicin and thapsigargin induced chaperone and CHOP expression, demonstrating the ability of cultured cortical neurons to develop an UPR (Figures 6D,E). These results suggest that mdivi-1 facilitates the activation of the ISR in neurons in an ER stress-independent manner.

### Mdivi-1 Modulates Mitochondrial [Ca^2+^] and Function in Neurons

**Figure 7:**
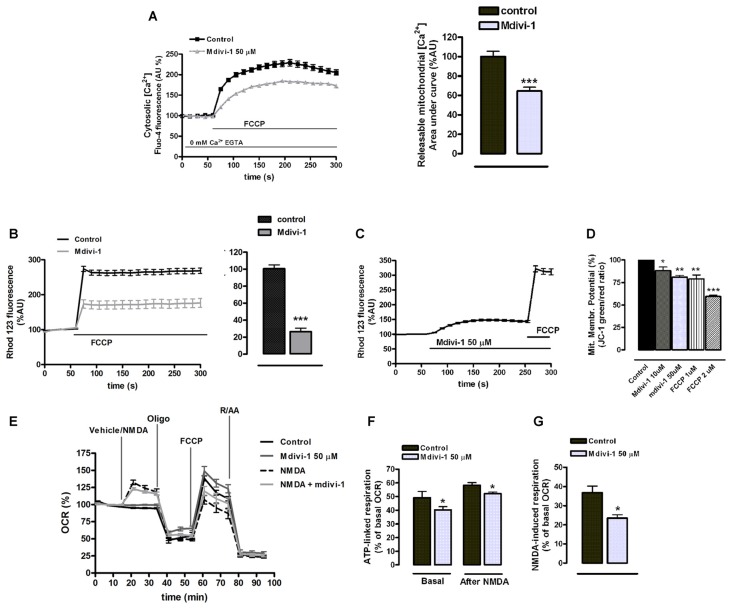
Mdivi-1 regulates mitochondrial [Ca^2+^], membrane potential and respiration. **(A)** Neurons were incubated with Fluo-4 in a Ca^2+^-free medium containing EGTA and exposed to FCCP (1 μM) in the presence or absence of mdivi-1 (50 μM). Resulting cytosolic Ca^2+^ increase was measured to determine FCCP-releasable [Ca^2+^]_mit_. Traces represent normalized means ± SEM of control (*n* = 74) and mdivi-1-treated cells (*n* = 77) from at least three independent cultures. ***p < 0.001 compared to control cells, Mann-Whitney U test. **(B,C)** Cells were incubated with quenching concentrations (5 μM) of rhodamine 123 and exposed to FCCP and the increase in fluorescence was measured to determine mitochondrial membrane potential. **(B)** Traces represent normalized means ± SEM of control (*n* = 86) and 10 μM of mdivi-1-treated (*n* = 55) cells from at least three independent cultures. ***p < 0.001 compared to control cells, Mann-Whitney U test. **(C)** Traces represent normalized means ± SEM of rhodamine 123 fluorescence from neurons sequentially treated with mdivi-1 (50 μM) and FCCP (1 μM).** (D)** Mitochondrial membrane potential was measured using JC-1 fluorescent dye 45 min after 10 μM (*n* = 5) and 50 μM (*n* = 4) of mdivi-1 application or 15 min after 1 μM (*n* = 4) and 2 μM (*n* = 3) of FCCP addition. Data represent normalized means ± SEM of the JC-1 red/green fluorescence ratio. *p < 0.05, **p < 0.01, ***p < 0.001 compared with control (untreated cells), paired Student’s *t*-test. **(E–G)** Primary neurons in the presence or absence of mdivi-1 (50 μM, 1 h) were exposed to vehicle or NMDA (30 μM), oligomycin (2 μM), FCCP (1 μM) and rotenone plus antimycin A (both 0.5 μM) and mitochondrial oxygen consumption rate (OCR) measured. Traces represent normalized means ± SEM of *n* = 5 experiments. ATP-linked respiration **(F)** and NMDA-stimulated respiration **(G)** are represented as a percentage of the basal OCR. *p < 0.05 compared with control (untreated cells or NMDA alone), paired Student’s *t*-test.

ER tubules are physically and functionally connected to mitochondria in terms of Ca^2+^ signaling (Raturi and Simmen, 2013). Since mdivi-1 significantly regulated ER Ca^2+^ stores we next analyzed the impact of this drug in mitochondrial Ca^2+^ storage and bioenergetics. To study a possible effect of mdivi-1 on resting [Ca^2+^]_mit_, we measured [Ca^2+^]_cyt_ upon addition of FCCP in the absence of extracellular Ca^2+^, which is indicative for [Ca^2+^]_mit_ (Brocard et al., 2001). Interestingly, we found that mdivi-1 significantly reduced FCCP-induced [Ca^2+^]_cyt_ increase from 228.9 ± 7.7% to 184.5 ± 4.3%, indicating that it partially depleted mitochondrial Ca^2+^ store in resting conditions (Figure 7A). Next, we obtained semi-quantitative measurements of mitochondrial membrane potential in control and mdivi-1 treated neurons using live cell imaging of Rh 123 fluorescent dye under “dequenching” conditions (Corona and Duchen, 2014). Addition of FCCP to dequench Rh 123 increased cytoplasmic fluorescence to 274.1 ± 7.6% over baseline (100%) in control cells. In the presence of pre-incubated (10 μM, 1 h) mdivi-1, FCCP-induced Rh 123 fluorescence increase was reduced to 173.5 ± 12.1% over baseline (Figure 7B). Addition of mdivi-1 alone triggered a fast increase in Rh 123 fluorescence to 148.1 ± 3.2% over baseline (100%), indicating that the drug acutely depolarized the mitochondrial membrane (Figure 7C). Alternatively, analysis of mitochondrial membrane potential by JC-1 fluorescence probe revealed a dose-dependent mitochondrial membrane depolarization to 88.2 ± 4% and 80.8 ± 1.9% of control (untreated cells, 100%) by 10 μM and 50 μM of mdivi-1, respectively. Responsiveness of JC-1 was determined by the addition of FCCP as a positive control, which reduced mitochondrial membrane potential to 78.9 ± 4.4% and 59.4 ± 1.6% of control at 1 μM and 2 μM, respectively (Figure 7D).

We next analyzed the effect of midivi-1 on mitochondrial respiration during excitotoxicity, since it was recently shown to reversibly inhibit mitochondrial electron transport chain at complex I (Bordt et al., 2017). After 1 h incubation, mdivi-1 reduced neuronal ATP-linked mitochondrial respiration from 49.2 ± 4.5% of cellular OCR (basal line, 100%) to 40.3 ± 2.3% in vehicle-treated cells (Figures 7E,F). In NMDA-treated neurons, excitotoxic insults induced an increase in the OCR of 36.7 ± 3.4% over basal line (100%), which was attenuated to 23.4 ± 1.8% in the presence of mdivi-1 (Figures 7E,G). The respiratory fraction linked to ATP production during NMDA receptor activation was reduced as well by mdivi-1 from 49.2 ± 4.6% of total OCR to 40.3 ± 2.3% (Figures 7E,F). These results are consistent with a moderate reduction of mitochondrial respiration by mdivi-1 that leads to a decreased mitochondrial potential and [Ca^2+^]_mit_.

### NMDA-Induced Mitochondrial Fission and Toxicity Are Independent of Drp1

**Figure 8:**
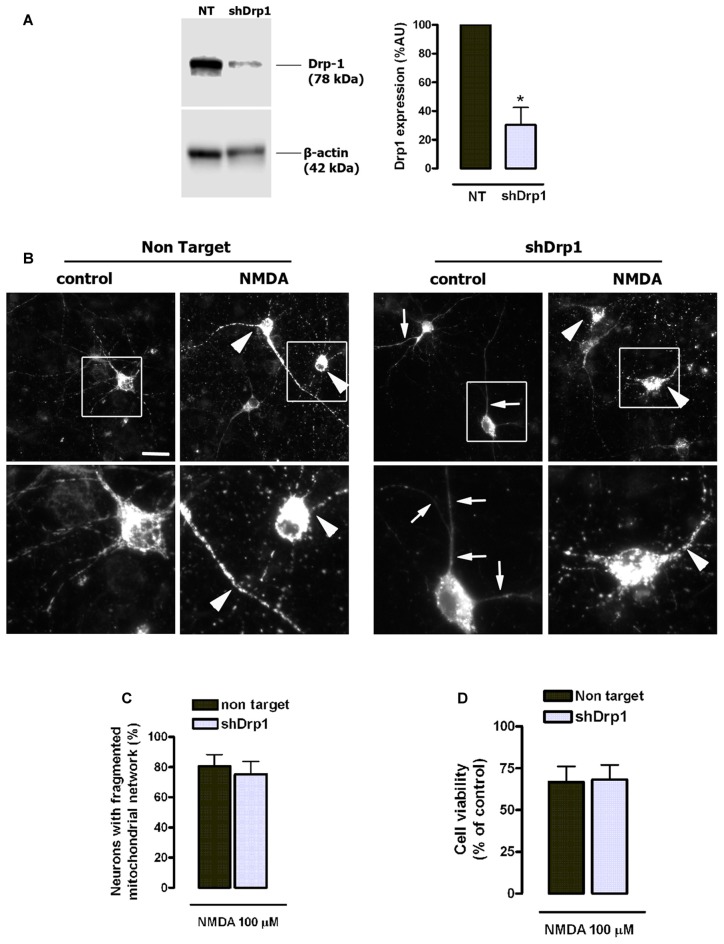
NMDA-induced mitochondrial fission and toxicity is not prevented by dynamin related protein 1 (Drp1) knockdown.** (A)** After lentiviral delivery of shRNA and puromycin selection cells were harvested for the detection of Drp1 by western blot. Drp1 signal was measured and normalized to β-actin values (*n* = 3). *p < 0.05, compared with cells expressing non target (NT) shRNA, paired student’s *t*-test.** (B)** Representative images of NMDA-induced mitochondrial fragmentation in shRNA expressing neurons. Neurons transfected with mitochondria-targeted 2mtD4cpv and infected with shRNA-carrying lentiviruses were exposed to 100 μM of NMDA for 30 min and fixed for mitochondrial morphology analysis. White boxes on top images correspond to zoomed fields (bottom row). Arrowheads indicate fragmented mitochondrial networks and arrows mark abnormally elongated mitochondrial network in Drp1-silenced neurons. Scale bar: 20 μM. **(C)** Mitochondrial morphology analysis was performed by the quantification of cells with fragmented mitochondrial network in neurons expressing NT shRNA (*n* = 179 cells) and shDrp1 (*n* = 167 cells). Paired Student’s *t*-test. **(D)** Neurons were treated as in **(B)** and 24 h later cell viability was assessed by the quantification of calcein-AM fluorescence (*n* = 3). Data represent means ± SEM of normalized calcein fluorescence values, paired Student’s *t* test.

Pharmacological and genetic inhibitors of Drp1 partially inhibit NMDA-induced mitochondrial fragmentation (Martorell-Riera et al., 2014), and Drp1/Mfn-independent mechanisms have been proposed to alternatively shape mitochondrial network (Rival et al., 2011; Stavru et al., 2013; Yamashita et al., 2016). Thus, we next analyzed the contribution of Drp1 to mitochondrial fragmentation and toxicity during NMDA-mediated excitotoxicity using a genetic and therefore more specific approach by Drp1 knockdown. Lentiviral delivery of shRNA reduced neuronal Drp1 expression to 30.3% ± 12.1% of control (100%, non target (NT) shRNA; Figure 8A), and induced an abnormally elongated mitochondrial network phenotype (Figure 8B). However, Drp1 knockdown did not attenuate either mitochondrial fission (Figures 8B,C) or cell death (Figure 8D) in NMDA-treated neurons, unveiling that neuroprotection provided by mdivi-1 in NMDA-induced excitotoxic conditions is mainly Drp1-independent.

## Discussion

### Effects of Mdivi-1 on Mitochondrial Fission and Excitotoxicity

Activation of ionotropic glutamate receptors in neurons induces fast and transient mitochondrial fragmentation through a Ca^2+^- and NMDA-dependent mechanism (Rintoul et al., 2003; Martorell-Riera et al., 2014). Consistent with those findings, we observed that incubation with NMDA or AMPA/CTZ caused fragmentation of the mitochondrial network in most cultured neurons within the first 30 min. The high degree of mitochondrial fission observed did not correlate with the extent of neuronal death, consistent with the generally accepted idea that mitochondrial fragmentation does not necessarily lead to cell death. Mdivi-1 was able to strongly inhibit mitochondrial fragmentation triggered by NMDA, in agreement with previous reports that suggested the involvement of Drp1 in excitotoxic mitochondrial fission (Grohm et al., 2012; Martorell-Riera et al., 2014). However, mdivi-1 failed to block AMPA/CTZ-induced mitochondrial fission, suggesting that mdivi-1 may have alternative targets different to the fusion/fission machinery. To date, most of the studies reporting a neuroprotective effect of mdivi-1 against brain ischemia or excitotoxicity provided evidence of anti-apoptotic activity of the drug (Grohm et al., 2012; Zhang et al., 2013; Zhao et al., 2014), consistent with the role of Drp1 in programmed cell death (Frank et al., 2001). However, our results suggest that mdivi-1 also exerts a robust protection against NMDA-induced necrotic-like neuronal death, according to its ability to reduce early LDH release and to strongly inhibit calpain activation. LDH release was reduced by mdivi-1 as early as 1 h after the excitotoxic stimulus, whereas addition of the inhibitor immediately after NMDA failed at reducing delayed cell death measured 24 h later. In addition, protection against increasing concentrations of NMDA showed a strong correlation with the reduction in calpain activity observed in the presence of mdivi-1. Calpains are Ca^2+^-activated cysteine proteases that play a pivotal role in the induction of necrosis in ischemic and excitotoxic neuronal injury (Wang, 2000; Lai et al., 2014). In particular, our previous results demonstrated that NMDA triggered calpain activation with no significant caspase-3 activity in cultured cortical neurons (Ruiz et al., 2014), as described earlier in hippocampal neurons (Lankiewicz et al., 2000).

### Effects of Mdivi-1 on Excitotoxic [Ca^2+^]_cyt_ Overload

Inhibition of calpain activation also correlated with the reduction of the NMDA-induced [Ca^2+^]_cyt_ increase observed in the presence of mdivi-1. Interestingly, mdivi-1 strongly reduced [Ca^2+^]_cyt_ signals induced by KA, AMPA and VGCC activation, showing that the effect of this drug on neuronal Ca^2+^ signaling is not NMDAR specific. However, mdivi-1 did not reduce desensitizing AMPA/CTZ-induced [Ca^2+^]_cyt_ overload and downstream calpain activation, while it was to a lesser extent protective. The results obtained in the presence of CTZ revealed that: (i) mdivi-1 does not act as a calpain inhibitor; and (ii) that it may additionally protect against excitotoxicity independently of its effects on the early [Ca^2+^]_cyt_ rise. Finally, since mdivi-1 failed at inhibiting AMPA/CTZ-induced mitochondrial fission, these results strongly suggest as well that the inhibition of NMDA-induced mitochondrial fission provided by mdivi-1 depends on its effects on [Ca^2+^]_cyt_ overload during excitotoxicity, rather than to its direct effect on Drp1.

### Effects of Mdivi-1 on ER Ca^2+^ Store and Mitochondrial Function

Anti-apoptotic Bcl-2 protein reduces [Ca^2+^]_ER_ (Foyouzi-Youssefi et al., 2000; Pinton et al., 2000; Palmer et al., 2004), providing cytoprotection against apoptosis (Pinton et al., 2001). In agreement with this concept, pro-apoptotic Bax contributes to Ca^2+^ crosstalk between ER and cytosol and its downregulation protects neurons against excitotoxicity (D’Orsi et al., 2015). Paradoxically, optimal Ca^2+^ levels are crucial for ER correct function and rapid and complete depletion of ER Ca^2+^ stores leads to ER stress and apoptosis (Verkhratsky and Petersen, 2002). In the current study, we show with two different imaging approaches that ER Ca^2+^ was partially depleted by mdivi-1, with no significant evidence of ER stress induction and neuronal death. However, we found that mdivi-1 enhanced eIF2α phosphorylation in both basal and excitotoxic conditions. Phosphorylation of eIF2α inhibits global protein synthesis and triggers the ISR, a cytoprotective cellular mechanism that has been previously linked to neuroprotection during excitotoxicity (Sokka et al., 2007; Ruiz et al., 2014).

Another key finding of this study is that mdivi-1 lowers mitochondrial membrane potential, [Ca^2+^]_mit_ and respiration. We observed that mdivi-1 reduced resting [Ca^2+^]_mit_, indicating that the lowered [Ca^2+^]_ER_ was unlikely a consequence of an enhanced Ca^2+^ export into mitochondria. Importantly, mdivi-1 depolarized the mitochondrial membrane, which could in part explain the reduced matrix free [Ca^2+^], since Ca^2+^ accumulation inside the mitochondria is membrane-potential dependent (Drago et al., 2011). Consistent with the drop in mitochondrial membrane potential, in the presence of mdivi-1 neurons showed a reduced mitochondrial respiration, which was not sufficient to induce an ATP depletion during NMDAR activation All these observations are in agreement with a recent study that demonstrated that mdivi-1 reversibly inhibits neuronal electron transport chain at complex I independently of Drp1, leading to a modulation of mitochondrial ROS production that may provide cytoprotection (Bordt et al., 2017). Our results suggest that mild inhibition of respiration and mitochondrial membrane depolarization triggered by mdivi-1 reduces excitotoxic injury, as previously observed with respiratory chain inhibitors or uncouplers. It was shown that under conditions of mitochondrial membrane depolarization and ATP availability glutamate ionotropic receptor-induced Ca^2+^ overload is reduced (Castilho et al., 1998; Rego et al., 2001), which could explain the effect of mdivi-1 on [Ca^2+^]_cyt_ signals triggered by NMDA, AMPA and KA. On the other hand, regardless of the extent of the [Ca^2+^]_cyt_ increase, mdivi-1 attenuated the [Ca^2+^]_mit_ rise triggered by both NMDA and AMPA/CTZ, an effect that can be reproduced by uncouplers and electron transport chain inhibitors and that attenuates excitotoxicity (Stout et al., 1998). Thus, we propose that the mild inhibition of respiration provided by mdivi-1 results in: (i) a broad inhibitory effect on plasma membrane glutamate ionotropic receptors; and (ii) a reduced mitochondrial Ca^2+^ overload during excitotoxicity.

### Contribution of Drp1 to NMDA-Induced Excitotoxicity

[Ca^2+^]_cyt_ increase results in Drp1 activation that leads to mitochondrial fragmentation (Cribbs and Strack, 2007; Cereghetti et al., 2008). However, it is not known to what extent Drp1 contributes to excitotoxicity. Similarly to a previously mentioned report (Bordt et al., 2017) our study suggests that the fast and robust effects of mdivi-1 on intracellular [Ca^2+^] and mitochondrial function during excitotoxicity are independent of Drp1 inhibition. This assumption was further supported by the lack of reduction of NMDA-induced mitochondrial fission and toxicity after lentiviral Drp1 knockdown. In agreement with these findings, previous studies reported that dominant mutant Drp1^K38A^ was unable to inhibit mitochondrial fission and neuronal death triggered by necrotic glutamate stimuli (Young et al., 2010) and NMDA (Barsoum et al., 2006). Moreover, Drp1 inhibition by mdivi-1 or genetic tools partially reduced mitochondrial fission during excitotoxicity which suggests a mechanism independent of Drp1 (Martorell-Riera et al., 2014).

Thus, further research is needed to reveal the molecular mechanisms involved in excitotoxic early mitochondrial fission, and exhaustive controls should be carried out when using mdivi-1 as a Drp1 inhibitor in conditions of intracellular Ca^2+^ homeostasis disruption in neurons.

**Figure 9:**
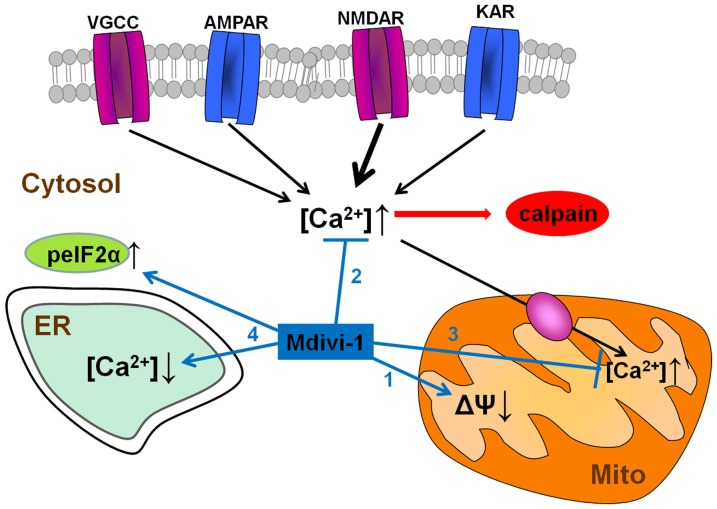
Neuroprotective pathways of mdivi-1. Mdivi-1 induces a moderate inhibition of mitochondrial respiration, and a drop in mitochondrial membrane potential (1). It also attenuates cytosolic Ca^2+^ overload due to activation of VGCCs and glutamate ionotropic receptors, and consequently, downstream NMDAR-dependent calpain activation (2). Mdivi-1-induced mitochondrial depolarization reduces mitochondrial Ca^2+^ uptake and therefore prevents excitotoxic [Ca^2+^]_mit_ overload (3). Mdivi-1 partially depletes ER Ca^2+^ store and enhances peIF2α phosphorylation (ISR; 4), events that provide cytoprotection against a variety of stress conditions.

In summary, we describe here that mdivi-1 strongly protects against NMDA-induced excitotoxicity by modulating mitochondrial function and intracellular Ca^2+^ signaling through Drp1-independent mechanisms (Figure 9). To our best knowledge, this is the first report demonstrating that mdivi-1 protects against excitotoxic Ca^2+^ overload and necrotic cell death, a feature that may expand its therapeutic potential for the treatment of brain diseases in which glutamate receptor overactivation is involved, such as ischemic brain injury and Alzheimer’s disease.

## Author Contributions

AR: designed and performed the experiments and drafted the manuscript. EA and CM: designed the project and experiments, helped with the interpretation of the results and critically revised the manuscript. All authors have read and approved of the manuscript.

## Conflict of Interest Statement

The authors declare that the research was conducted in the absence of any commercial or financial relationships that could be construed as a potential conflict of interest.
